# Transcription Factor MAX Regulates Liver Cancer Cell Growth, Migration, Invasion, and Epithelial–Mesenchymal Transition by Promoting SF3A3 Expression

**DOI:** 10.14740/wjon2783

**Published:** 2026-06-25

**Authors:** Gui Zhou, Mei Yu Dai, Xiang Chen, Li Ming Liu, Fa Quan Lin

**Affiliations:** a Key Laboratory of Clinical Laboratory Medicine of Guangxi Department of Education, Department of Clinical Laboratory, the First Affiliated Hospital of Guangxi Medical University, Nanning 530021, China; b Center For Reproductive Medicine, the Fourth Affiliated Hospital of Guangxi Medical University, Liuzhou 545005, China; c Medical Science Laboratory, the Fourth Affiliated Hospital of Guangxi Medical University, Liuzhou 545005, China; d Liuzhou Key Laboratory of Molecular Diagnosis, Affiliated Liutie Central Hospital of Guangxi Medical University, Liuzhou 545007, China; e Gui Zhou and Mei Yu Dai contributed equally to this study.

**Keywords:** Hepatocellular carcinoma, MAX, SF3A3, Alternative splicing, Transcription factor, Cancer stemness, Therapeutic target, Epithelial–mesenchymal transition

## Abstract

**Background:**

The regulatory relationship between transcription factor MAX and splicing factor SF3A3 in hepatocellular carcinoma (HCC) is unknown. We investigated whether MAX directly regulates SF3A3 and their functional role in HCC progression.

**Methods:**

MAX and SF3A3 expression were analyzed in The Cancer Genome Atlas Liver Hepatocellular Carcinoma (TCGA-HCC) dataset (UALCAN, GEPIA) and validated in 33 paired HCC and adjacent non-tumor tissues by quantitative reverse transcription polymerase chain reaction (qRT-PCR). Expression and prognostic significance were assessed across cancer stages, grades, and nodal status. Functional roles were evaluated in Hep3B (high expression) and PLC/PRF/5 (low expression) cells using shRNA-mediated knockdown and overexpression, respectively. Cell proliferation (cell counting kit-8, colony formation), migration (wound healing, Transwell), invasion (Matrigel Transwell), and epithelial–mesenchymal transition (EMT) (Western blot for E-cadherin, N-cadherin, and vimentin) were assessed. Mechanistic studies included chromatin immunoprecipitation (ChIP)-quantitative polymerase chain reaction (qPCR), luciferase reporter assays with site-directed mutagenesis, and Myc inhibition. *In vivo* tumor growth was evaluated using a xenograft mouse model.

**Results:**

MAX and SF3A3 were overexpressed in HCC tissues compared to normal liver, and high expression was correlated with reduced patient survival, advanced cancer stage, higher tumor grade, and nodal metastasis. A significant positive correlation between MAX and SF3A3 expression was observed. Functional assays demonstrated that MAX or SF3A3 overexpression promoted HCC cell proliferation, migration, invasion, and EMT, while knockdown suppressed these phenotypes. MAX directly bound the SF3A3 promoter (P3 E-box) and activated its transcription in a Myc-dependent manner. Overexpression of MAX or SF3A3 promoted malignant phenotypes, while SF3A3 knockdown reversed MAX-driven oncogenic effects *in vitro* and reduced tumor growth *in vivo.*

**Conclusion:**

This study establishes a novel MAX-SF3A3 regulatory axis in which MAX directly binds the SF3A3 promoter and activates its transcription in a Myc-dependent manner, driving HCC cell proliferation, migration, invasion, EMT, and tumor growth. Targeting the MAX-SF3A3 axis represents a potential therapeutic strategy for HCC.

## Introduction

Hepatocellular carcinoma (HCC) is a primary malignant tumor that originates from hepatocytes. It is the fourth leading cause of cancer-related deaths worldwide, accounting for approximately 800,000 deaths annually and posing a major threat to public health [1, 2]. The geographical distribution of HCC cases shows regional disparities, with the morbidity and mortality rates being notably higher in East Asia, where there is a high prevalence of hepatitis B virus (HBV) infection [3]. The median survival of untreated patients with HCC is only a few months [4].

Various treatment modalities are available for HCC, including surgical resection, liver transplantation, and targeted therapies such as sorafenib and lenvatinib [5, 6]. Patients in whom HCC is detected and treated early have a relatively good prognosis; however, treatment is challenging in patients with intermediate- to advanced-stage HCC [7]. Existing studies highlight the importance of early detection and treatment of HCC and emphasize the crucial roles of HBV infection and other factors in the development of HCC [8].

Tumorigenesis is a complex process that can be influenced by various factors, including genetic mutations, environmental stimuli, and lifestyle choices [9]. Among these factors, aberrant splicing has been shown to play a crucial role, particularly in the progression of cancer [10, 11]. Splicing is a fundamental process that allows for the diversification of the proteome from a limited number of genes, and its dysregulation can lead to the production of abnormal proteins that contribute to cancer development [12].

Understanding the intricate relationship between abnormal splicing patterns and tumorigenesis is important for the advancement of novel therapeutic interventions [13]. Recent studies have highlighted the crucial role of the core splicing factor SF3A3 in the regulation of alternative mRNA splicing, a process that enables the generation of multiple protein isoforms from a single gene [14, 15].

Overactivated Myc signaling increases the expression of SF3A3 [16]. This overexpression can have profound effects on cellular metabolism, leading to metabolic reprogramming that supports the survival and proliferation of cancer cells. Furthermore, the modulation of SF3A3 expression can affect stemness-related characteristics, which are associated with the self-renewal and differentiation potential of cells and are often hijacked in cancer to promote tumor initiation and progression [16].

The interaction between Myc and Myc-associated factor X (MAX) is crucial for the activation of the oncogenic potential of Myc [17, 18]. The Myc-MAX heterodimer can bind to DNA and regulate the transcription of genes involved in cell cycle progression, metabolism, and apoptosis [18, 19]. This regulatory network is often dysregulated in cancer, leading to uncontrolled cell growth and resistance to cell death, which are hallmarks of malignancy [19].

Given that SF3A3 can be upregulated by activated Myc signaling, we hypothesized that MAX might have a regulatory effect on SF3A3 in the development of liver cancer. However, to date, no study has directly investigated whether MAX binds to the SF3A3 promoter or transcriptionally regulates its expression in any cancer type. The molecular mechanism linking MAX to splicing factor dysregulation in HCC remains largely unexplored. In this study, we evaluated the expression of the MAX and SF3A3 genes in liver cancer and investigated their roles in the development of liver cancer through loss- and gain-of-function assays, with a particular focus on establishing whether MAX directly regulates SF3A3 transcription, thereby clarifying the mechanisms through which both MAX and SF3A3 contribute to the initiation and progression of liver cancer. To our knowledge, this study provides the first demonstration of direct MAX binding to the SF3A3 promoter in HCC, or indeed in any cancer context, thereby unveiling a novel transcriptional regulatory mechanism for splicing factors.

## Materials and Methods

### Bioinformatic analysis

To evaluate the expression levels of SF3A3 and MAX in liver cancer, gene expression data were extracted from the TCGA-HCC dataset from UALCAN [20]. The expression levels of both factors were compared between normal liver and primary liver tumor tissues. The analysis was stratified by cancer stages, tumor grades, nodal metastasis status, and histological subtypes to assess the correlation between SF3A3 or MAX expression and clinical outcomes. Furthermore, the correlation between MAX and SF3A3 was analyzed using the GEPIA database [21]. The JASPAR database [22], a resource of transcription factor-binding profiles, was queried using the MAX matrix profile MA0058.4 to predict potential transcription factor-binding sites within the SF3A3 promoter. Position weight matrix (PWM) scores were calculated for each putative binding site using the JASPAR scoring function, with higher scores indicating greater similarity to the consensus MAX-binding motif (CACATG). This *in silico* analysis provided a list of candidate binding sites, which were selected for further experimental validation.

### Measurement of MAX and SF3A3 in clinical tissue samples

A total of 33 paired HCC tissues and adjacent non-tumor liver tissues were obtained from patients who underwent surgical resection at the First Affiliated Hospital of Guangxi Medical University between December 2023 and September 2024. The cohort included 25 males and eight females, with an age range of 38–74 years (mean age: 55.6 ± 10.4 years). The inclusion criteria included: (1) patients with primary HCC confirmed by histopathological examination; (2) patients who underwent curative surgical resection; and (3) availability of paired tumor and adjacent non-tumor tissues. The exclusion criteria included: (1) patients who received chemotherapy, radiotherapy, or immunotherapy prior to surgery; (2) patients with other concurrent malignancies; and (3) tissue samples with insufficient RNA quality for analysis. All diagnoses were confirmed by histopathological examination. None of the patients had received preoperative chemotherapy or radiotherapy. Tissue specimens were immediately frozen in liquid nitrogen after resection and stored at −80 °C until RNA extraction. The RNA levels of MAX and SF3A3 in tissue samples were measured using quantitative reverse transcription polymerase chain reaction (qRT-PCR) method.

### Chromatin immunoprecipitation

Chromatin immunoprecipitation (ChIP) assay was performed to validate the interaction between MAX and the SF3A3 promoter. Hep3B and PLC/PRF/5 cells were cross-linked with formaldehyde to fix protein–DNA interactions and sonicated to shear the chromatin into smaller fragments. An anti-MAX antibody (#ab185913, Abcam, Cambridge, UK) was used to precipitate MAX-bound DNA fragments, with non-specific IgG serving as a negative control. The immunoprecipitated DNA was purified and quantified via quantitative polymerase chain reaction (qPCR) using primers flanking the predicted MAX-binding sites on the SF3A3 promoter. Enrichment was calculated as percentage of input DNA. Normal rabbit IgG (Cat# 2729, Cell Signaling Technology, Danvers, MA, USA) was used as a negative control. Each ChIP experiment was performed in triplicate with independent biological replicates. The ChIP-qPCR primer sequences and amplicon details for the SF3A3 promoter are provided in Supplementary Material 1 (wjon.elmerpub.com).

### Luciferase reporter assay

The SF3A3 promoter region, including the three predicted MAX-binding sites, was cloned into the pGL3-basic luciferase reporter vector (Cat# E1751, Promega, Madison, WI, USA). For mutation analysis, the core MAX-binding sequence (CACATG) at site P3 was mutated to ATCGAT using the QuikChange II Site-Directed Mutagenesis Kit (Cat# 200523, Agilent Technologies, Santa Clara, CA, USA). Hep3B and PLC/PRF/5 cells were co-transfected with the luciferase reporter construct (200 ng), a MAX-overexpression vector or a short hairpin RNA (shRNA) targeting MAX (100 ng), and the Renilla luciferase control vector pRL-TK (10 ng, Cat# E2241, Promega) using Lipofectamine 3000 (Thermo Fisher). After 48 h, the cells were lysed and luciferase activity was measured using a luciferase assay kit (#E6110, Promega, Madison, WI, USA) according to the manufacturer’s protocol. The luciferase activity was normalized to protein concentration and expressed as a fold change relative to the control. Each experiment was performed in triplicate with three independent replicates.

### Cell culture and establishment of stable cell lines

The 293T (No. CRL-3216), Hep3B (No. HB-8064), SK-Hep1 (No. HTB-52), SNU-182 (No. CRL-2235), PLC/PRF/5 (No. CRL-8024), and THLE-2 (No. CRL-2706) cell lines were obtained from ATCC and cultured according to the manufacturer’s guidelines at 37 °C with 5% CO_2_. Hep3B, PLC/PRF/5, and 293T cells were cultured in DMEM (Cat# 11965092, Gibco, Thermo Fisher Scientific, Waltham, MA, USA) supplemented with 10% fetal bovine serum (FBS, Cat# 10099141, Gibco, Thermo Fisher Scientific). THLE-2 cells were maintained in bronchial epithelial growth medium (BEGM) (Cat# CC-3170, Lonza, Basel, Switzerland) supplemented with specific growth factors according to the manufacturer’s instructions.

We overexpressed human MAX (Gene name: MYC associated factor X, NCBI Reference Sequence: NM_001271068.2) and SF3A3 (Gene name: splicing factor 3a subunit 3, NCBI Reference Sequence: NM_001320830.2). To establish stable cell lines with gene overexpression or knockdown, the entire target gene or an shRNA targeting the gene was synthesized (Genescript, Piscataway, NJ, USA) and cloned into a lentiviral core vector containing a puromycin resistance gene. The shRNA sequences used were as follows: sh-MAX: Target Sequence (21nt): CCAAACCAGGTCAGCTATCAA; Oligo: forward Oligo: 5'-CCGG CCAAACCAGGTCAGCTATCAA CTCGAG TTGATAGCTGACCTGGTTTGG TTTTT G-3'; reverse Oligo: 5'-AATT C AAAAA TTGATAGCTGACCTGGTTTGG CTCGAG CCAAACCAGGTCAGCTATCAA-3'; sh-SF3A3: Target Sequence (21nt): GCTGAAGAAGCTCAATGGAAA; Oligo: forward Oligo: 5'-CCGG GCTGAAGAAGCTCAATGGAAA CTCGAG TTTCCATTGAGCTTCTTCAGC TTTTT G-3'; reverse Oligo: 5'-AATT C AAAAA TTTCCATTGAGCTTCTTCAGC CTCGAG GCTGAAGAAGCTCAATGGAAA-3'. The lentiviral core (backbone) vector used for overexpression was pLVX-Puro (Takara Bio, #632164) and the vector for shRNA constructs was pLKO.1 (Addgene, plasmid #8453). Overexpression negative control (OE-NC) was the empty pLVX-Puro vector, and short hairpin RNA negative control (sh-NC) was a lentiviral vector encoding a scrambled shRNA sequence (5'-TTCTCCGAACGTGTCACGT-3') with no homology to any human gene. The packaging plasmids were co-transfected into 80% confluent 293T cells in a T25 cell flask using the PEIpro transfection reagent (#115-100, Polyplus, New York, NY, USA). After 48 h, the culture supernatant was collected and centrifuged at 2,000 g to remove cell debris and obtain viral particles. For cell transfection, target cells were seeded in a six-well plate and cultured for 24 h. Subsequently, 500 µL of the viral solution was added to the cells, and the culture was continued. After 24 h, the culture medium was replaced with a fresh medium. After 48 h, puromycin (#540222, Millipore, St. Louis, MO, USA) at a final concentration of 5 µg/mL was added to the culture medium, and the culture was continued to obtain stably transfected cell lines. Transfection or infection efficiency was determined by measuring the mRNA and protein expression levels of the target genes using quantitative RT-PCR and Western blotting, respectively, 72 h post-transfection or infection.

### Cell migration and invasion assays

Cell migration was assessed using a wound healing assay. Briefly, a sterile pipette tip was used to scrape a confluent cell monolayer to create a cell-free gap. The ability of the cells to migrate into the gap zone was monitored over time, and images were captured at regular intervals (0, 24 h) using a phase-contrast microscope. The wound healing rate was quantified by measuring the gap area at different time points (0, 24 h) and calculating the healing rate.

Transwell invasion assay was used to assess the functional roles of MAX and SF3A3 in HCC cell invasion. Briefly, cells were seeded in Matrigel-coated Transwell inserts (#3422, Corning, NY, USA), and their ability to invade through the extracellular matrix was evaluated by counting the number of cells that had migrated to the lower side of the insert membrane after a pre-determined duration. The invasive cells were fixed and stained before being counted under a microscope.

### Myc inhibition experiments

To assess the role of Myc in MAX-mediated SF3A3 transcription, two complementary approaches were used. First, Hep3B and PLC/PRF/5 cells were treated with MAX-overexpressing plasmid and the Myc inhibitor 10058-F4 (#475956, Sigma-Aldrich) at concentrations of 10 µM for 24 h. Second, cells were transfected with MAX-overexpressing plasmid using Lipofectamine 3000 (Cat# L3000015, Thermo Fisher) and Myc-specific small interfering RNA (siRNA) (si-Myc, #sc-29226, Santa Cruz Biotechnology) using Lipofectamine RNAiMAX (#13778150, Thermo Fisher Scientific). The siRNA targeting human MYC (si-Myc) had the following sequence: sense: 5'-CCAACAGGAACUAUGACCUCGACUAA-3'; anti-sense: 5'-UAGUCGAGGUCAUAGUUCCUGUUGG-3'. Cells were seeded in six-well plates (2 × 10^5^ cells/well) and transfected with 100 nM siRNA at 37 °C for 48 h. After 48 h, cells were harvested for protein extraction, and SF3A3 expression was analyzed by Western blotting. The siRNA efficiency was determined by measuring the mRNA and protein expression levels of the target genes using quantitative RT-PCR and Western blotting, respectively, 48 h post-transfection.

### Western blotting

Cells were lysed using RIPA buffer (Cat# P0013B, Beyotime, Shanghai, China) supplemented with a protease inhibitor cocktail (Cat# 04693116001, Roche, Basel, Switzerland). Proteins were separated using SDS-PAGE. After the separated proteins were transferred onto a membrane, the membrane was incubated with specific primary antibodies against E-cadherin (#3195, Cell Signaling Technology, Danvers, MA, USA), N-cadherin (#13116, Cell Signaling Technology, Danvers, MA, USA), and vimentin (#5741, Cell Signaling Technology, Danvers, MA, USA). GAPDH (Cat# 5174, Cell Signaling Technology) was used as an internal control. Subsequently, the membrane was incubated with anti-rabbit IgG (Cat# 7074, Cell Signaling Technology) or anti-mouse IgG (Cat# 7076, Cell Signaling Technology) conjugated to horseradish peroxidase. Immunoreactive bands were visualized using a chemiluminescence detection system (ECL Chemiluminescent Substrate, Cat# 32106, Thermo Fisher Scientific), and the intensities of the bands were quantified using densitometry to assess relative protein expression levels.

### Quantitative reverse transcription polymerase chain reaction

Total RNA was extracted from cells or tissues using TRIzol Reagent (Cat# 15596026, Invitrogen, Carlsbad, CA, USA), and complementary DNA (cDNA) was synthesized using a reverse transcription kit (#RR037Q, Takara, China). Subsequently, qPCR was performed using SYBR Green Master Mix (#A25741, Thermo Fisher Scientific, Waltham, MA, USA) on a QuantStudio 5 real-time PCR system (Applied Biosystems, Foster City, CA, USA) with gene-specific primers for MAX (forward: 5'-AGTGAGGAGGTGGTTCTTGC-3', reverse: 5'-GCTCATTTCCTACGGCCCAG-3'), SF3A3 (forward: 5'- CCATGCAAGATATCTGTGTGCC-3', reverse: 5'-ACTCCACCAAGTTTTGTGCC -3'), and GAPDH (internal control; forward: 5'-AATGGGCAGCCGTTAGGAAA-3', Reverse: 5'-GCGCCCAATACGACCAAATC-3'). The relative RNA expression levels of target genes were calculated using the 2^-ΔΔCt^ method, allowing for the quantification of fold changes in gene expression.

### Colony formation assay

For colony formation assay, HCC cells were seeded in six-well plates at a low density (1,000 cells/well). After overnight incubation, the medium was replaced with a fresh complete growth medium. The medium was changed every 3 days for 10–14 days until the colonies became visible. The colonies were fixed with 10% formalin (Cat# HT501128, Sigma-Aldrich), stained with 0.5% crystal violet (Cat# C0775, Sigma-Aldrich), and counted manually. Only colonies consisting of ≥ 50 cells were considered, and the plating efficiency was calculated.

### CCK-8 assay

To evaluate cell proliferation over 1–5 days, cells were seeded in 96-well plates and cultured. On each day, cell counting kit-8 (CCK-8) reagent (#C0038, Beyotime, Shanghai, China) was added directly to the culture medium (10 µL per well), and the plate was incubated for 2 h at 37 °C. Absorbance at 450 nm was measured using an Epoch Microplate Spectrophotometer (BioTek, Winooski, VT, USA), which provided a daily assessment of cellular metabolic activity, indicative of cell proliferation.

### *In vivo* tumor growth assay

A nude mouse model of liver cancer was used to assess tumor growth *in vivo*. PLC/PRF/5 cells, overexpressing MAX (OE-MAX) with or without SF3A3 knockdown, were used to induce the formation of subcutaneous tumors in nude mice (BALB/c-nu/nu, 4–6 weeks, male) purchased from Laboratory Animal Center of Guangxi Medical University. The animals were housed under specific pathogen-free (SPF) conditions (temperature 22 ± 2 °C, humidity 50±10%, 12 h light/12 h dark cycle) with free access to sterile food and water. The PLC/PRF/5 cells were trypsinized, counted, and resuspended in a mixture of Matrigel Basement Membrane Matrix (Cat# 356234, Corning, NY, USA) and PBS at a concentration of 1 × 10^7^ cells/mL. A total of 100 µL of this cell suspension was subcutaneously injected into the right flank of each mouse. After the injection, the mice were monitored daily to assess general health and tumor development. Tumor size was measured using a caliper, and tumor volume (V) was calculated as (π/6) × (L × W^2^), where L is the length and W is the width of the tumor. The mice were randomly divided into five groups, with five mice per group, to ensure statistical relevance. The experiment spanned 5 weeks to allow for substantial tumor growth. Tumor growth was monitored weekly. Mice were monitored at least once daily for body weight, physical appearance, mobility, grooming, tumor burden, and any signs of pain or distress. Humane endpoints included a tumor burden > 1,500 mm^3^, weight loss exceeding 20%, severe lethargy, persistent hunched posture, or ulceration at the tumor site. When mice reached these endpoints, or at scheduled endpoints, they were euthanized by quick cervical dislocation under isoflurane anesthesia to ensure death. The tumors were excised and weighed to quantify tumor mass.

### Institutional Review Board approval

This clinical sample study was approved by the Ethics Committee of the First Affiliated Hospital of Guangxi Medical University (Approval No. 2023-E355-03). All experimental protocols were approved by the Ethics Committee of the First Affiliated Hospital of Guangxi Medical University (Approval No. 2024-E168-11), and all procedures conformed to institutional guidelines.

### Ethical compliance with human/animal study

This study was conducted in compliance with the ethical standards of the responsible institution on human subjects as well as with the Helsinki Declaration. The animal study was conducted in compliance with all the applicable institutional ethical guidelines for the care, welfare, and use of animals.

### Statistical analysis

The GraphPad Prism (version 9.0) software was used to ensure the accuracy and reliability of the results. Various analytical methods were used to assess differences under different experimental conditions. One-way analysis of variance (ANOVA) accompanied by the Bonferroni *post hoc* test was used to compare multiple groups, allowing for the detection of significant differences between each pair of groups. A paired, two-tailed *t*-test was used to compare gene expression between paired HCC tissues and adjacent non-tumor tissues. An unpaired, two-tailed *t*-test was used to compare the data of two independent groups (e.g., *in vitro* assays). A P-value < 0.05 was considered statistically significant.

## Results

### Expression of SF3A3 in HCC

**Figure 1:**
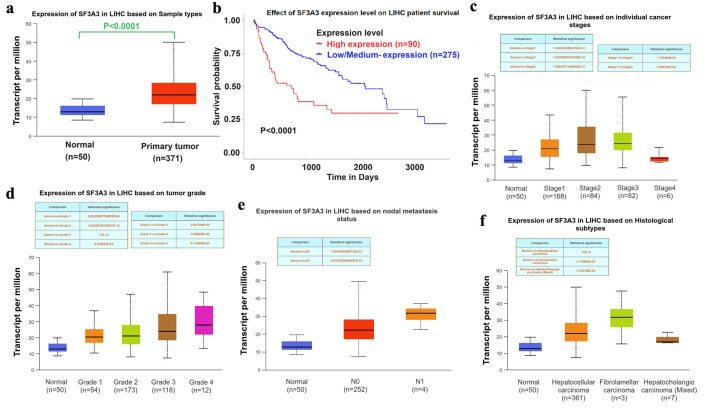
SF3A3 expression and clinical significance in hepatocellular carcinoma (HCC). (a) Expression of SF3A3 in primary LIHC tissues versus normal liver tissues (TCGA cohort). (b) Kaplan–Meier survival curves of LIHC patients stratified by SF3A3 expression. SF3A3 expression levels stratified by (c) cancer stage, (d) tumor grade, (e) nodal metastasis status, and (f) histological subtypes. Data are presented as mean ± SEM.

Gene expression data from the TCGA-HCC dataset were analyzed to evaluate the expression of SF3A3 in HCC. The results are presented in Figure 1. As shown in Figure 1a, the expression of SF3A3 was significantly higher in HCC tissues than in normal liver tissues, suggesting a potential role of SF3A3 in the pathogenesis of HCC. As shown in Figure 1b, higher expression levels of SF3A3 were correlated with shorter survival in patients with HCC, with the Kaplan–Meier curve demonstrating a significantly lower survival rate in the high-expression group (n = 90) than in the low/medium-expression group (n = 275) (P-value < 0.0001). When stratified by cancer stage, the expression of SF3A3 increased with advancing stages of HCC, from normal to stage 4, with the highest expression level being observed in patients with stage 4 disease (Fig. 1c). Similarly, when stratified by tumor grade, the expression of SF3A3 showed a consistent trend, indicating its potential association with tumor aggressiveness (Fig. 1d). When stratified by tumor grade, SF3A3 expression progressively increased from grade 1 to grade 3. Although expression in grade 4 appeared slightly lower than in grade 3, this difference was not statistically significant (P = 0.82), likely due to the smaller sample size (grade 4, n = 12) and greater variability in this group.

As shown in Figure 1e, the expression of SF3A3 significantly differed between patients with and without nodal metastasis. In addition, patients with different histological subtypes of HCC exhibited distinct expression patterns of SF3A3, indicating the presence of subtype-specific molecular characteristics and their influence on disease progression (Fig. 1f). These results provide a valuable basis for further investigation of SF3A3 as a potential biomarker and therapeutic target in HCC.

### Expression of MAX in HCC

**Figure 2:**
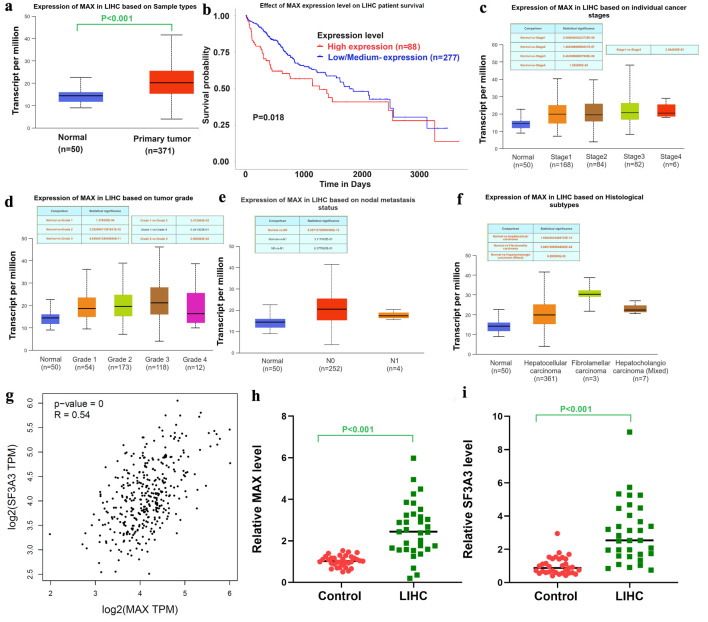
MAX expression and its correlation with SF3A3 in HCC. (a) Expression of MAX in primary LIHC tissues versus normal liver tissues (TCGA cohort). (b) Kaplan–Meier survival curves of LIHC patients stratified by MAX expression. MAX expression levels stratified by (c) cancer stage, (d) tumor grade, (e) nodal metastasis status, and (f) histological subtypes. (g) Correlation between MAX and SF3A3 expression in the TCGA-LIHC dataset. qRT-PCR analysis of MAX (h) and SF3A3 (i) mRNA levels in 33 paired HCC and adjacent normal liver tissues. Data are analyzed using a paired t-test and presented as mean ± SEM.

Given the upregulation of SF3A3 in HCC tissues, we investigated the expression of the transcription factor MAX in the TCGA-HCC dataset. The results showed that MAX was substantially upregulated in HCC tissues relative to normal liver tissues (Fig. 2a). As shown in the Kaplan–Meier curve in Figure 2b, patients in the high-MAX-expression group had poor survival. When stratified by cancer stage and tumor grade, the expression patterns of MAX were consistent with those of SF3A3. Specifically, the expression of MAX increased with disease progression (Fig. 2c–f). These findings collectively suggest that MAX is closely related to the malignant transformation and progression of HCC. Similarly to SF3A3, MAX expression did not significantly differ between grade 3 and grade 4 (P = 0.15), which may also be attributed to the limited sample size in the grade 4 group (n = 12). Compared with normal liver tissues, both SF3A3 and MAX were significantly upregulated across all tumor grades, supporting their overall association with HCC progression.

According to the GEPIA database, a significant linear correlation was observed between MAX and SF3A3 expression in HCC tissues (Fig. 2g). This correlation suggests a potential regulatory interplay between the two genes, which may play a crucial role in the pathogenesis of HCC. To validate the results of bioinformatic analysis, qRT-PCR was performed on 33 normal liver tissues and 33 HCC tissues. As shown in Figure 2h and i, the mRNA expression levels of MAX and SF3A3 were significantly higher in HCC tissues than in normal liver tissues, which was consistent with the results of bioinformatic analysis. Altogether, these results indicate a synergistic role of MAX and SF3A3 in the development of HCC, necessitating further investigation into the underlying mechanisms.

### Assessment of MAX and SF3A3 expression and functional roles of MAX and SF3A3 in HCC cell proliferation

**Figure 3:**
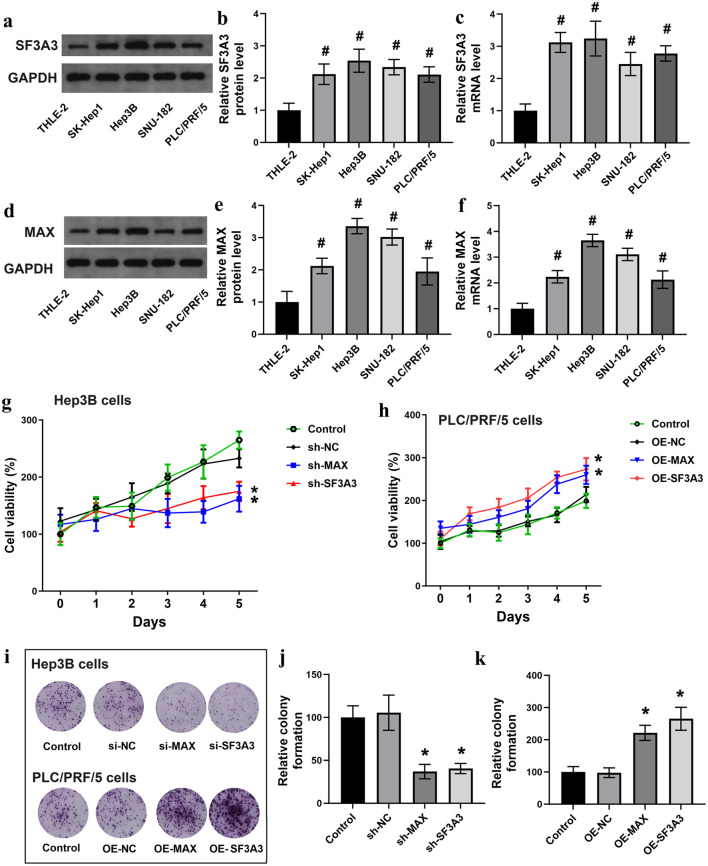
MAX and SF3A3 promote HCC cell proliferation *in vitro*. Baseline mRNA and protein expression of SF3A3 (a–c) and MAX (d–f) in the normal human liver cell line (THLE-2) and four HCC cell lines. CCK-8 assays evaluating cell proliferation over 5 days in Hep3B cells with MAX/SF3A3 knockdown (g) and PLC/PRF/5 cells with MAX/SF3A3 overexpression (h). (i)–(k) Colony formation assays for the indicated cell groups. Data are presented as mean ± SEM (n = 3). ^#^P < 0.05 compared to THLE-2 cells; *P < 0.05 compared to the respective control group.

To assess the functional roles of MAX and SF3A3 in HCC, we evaluated their expression levels in five cell lines *in vitro*. The results revealed notable differences in expression, with Hep3B cells exhibiting the highest expression and PLC/PRF/5 cells exhibiting the lowest expression of both SF3A3 (Fig. 3a–c) and MAX (Fig. 3d–f). In subsequent experiments, Hep3B cells were transfected with shRNAs to knock down MAX and SF3A3, whereas PLC/PRF/5 cells were transfected with plasmid constructs to overexpress the two genes. The effects of gene knockdown or overexpression on cell proliferation and proliferation were monitored over 5 days, with a focus on changes in cell proliferation and the number of cell colonies formed (Fig. 3g–k). Altogether, the results indicated that SF3A3 and MAX promoted the proliferation and survival of liver cancer cells.

### Function of MAX and SF3A3 in HCC cell migration, invasion, and EMT

**Figure 4:**
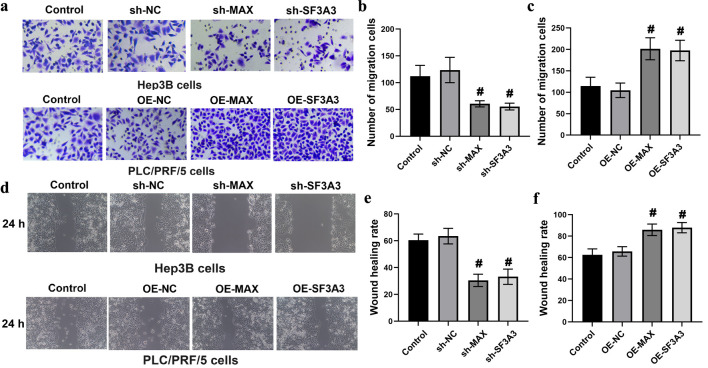
MAX and SF3A3 regulate HCC cell migration. (a)–(c) Transwell migration assays of Hep3B cells (knockdown) and PLC/PRF/5 cells (overexpression). (d)–(f) Wound healing assays evaluating the migration rate of the indicated cell groups at 0 and 24 h. Data are presented as mean ± SEM (n = 3). ^#^P < 0.01 compared to the respective control group.

We investigated the functional roles of MAX and SF3A3 in HCC cell migration and invasion, which are key hallmarks of cancer progression. Silencing MAX and SF3A3 in Hep3B cells significantly decreased the count of migratory cells, while overexpressing these two genes in PLC/PRF/5 cells markedly increased the number of cells with migratory ability (Fig. 4a–c). Similarly, wound healing assay showed that the wound healing rate of Hep3B cells with shRNA-mediated knockdown of MAX and SF3A3 was significantly lower than that of control cells. On the contrary, overexpression of MAX and SF3A3 in PLC/PRF/5 cells led to a remarkable increase in the wound healing rate (Fig. 4d–f).

**Figure 5:**
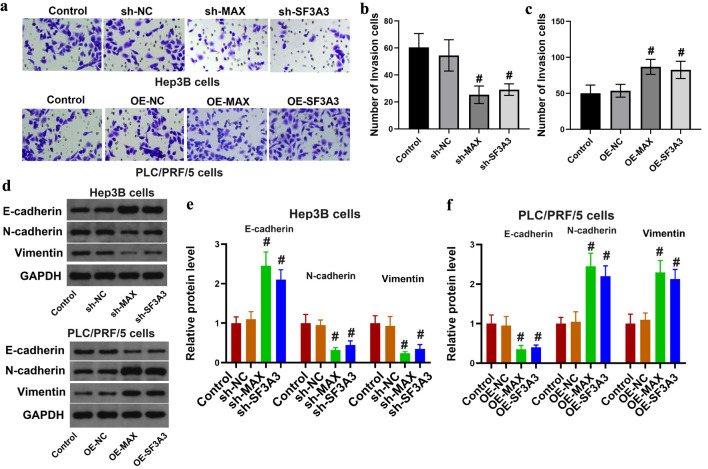
MAX and SF3A3 regulated liver cancer cell invasion and EMT. (a)–(c) Matrigel Transwell invasion assays of Hep3B cells (knockdown) and PLC/PRF/5 cells (overexpression). (d)–(f) Western blot analysis of EMT-related proteins (E-cadherin, N-cadherin, and vimentin) in the indicated cell groups. GAPDH was used as the loading control. Data are presented as mean ± SEM (n = 3). ^#^P < 0.05 compared to the respective control group.

In addition, knockdown of MAX and SF3A3 in Hep3B cells resulted in a significant reduction in the number of invasive cells, whereas overexpression of MAX and SF3A3 in PLC/PRF/5 cells led to a notable increase in the number of invasive cells (Fig. 5a–c). Furthermore, Western blotting was used to assess the expression of proteins associated with epithelial-mesenchymal transition (EMT). The results showed that knockdown of MAX and SF3A3 in Hep3B cells resulted in a significant change in the expression of EMT-related proteins, whereas overexpression of MAX and SF3A3 in PLC/PRF/5 cells led to a pronounced opposite change in the expression of these proteins (Fig. 5d–f). These results collectively suggest that the SF3A3 and MAX genes play a crucial role in HCC cell invasion, migration, and EMT.

### Mechanism of regulation of SF3A3 expression by MAX

**Figure 6:**
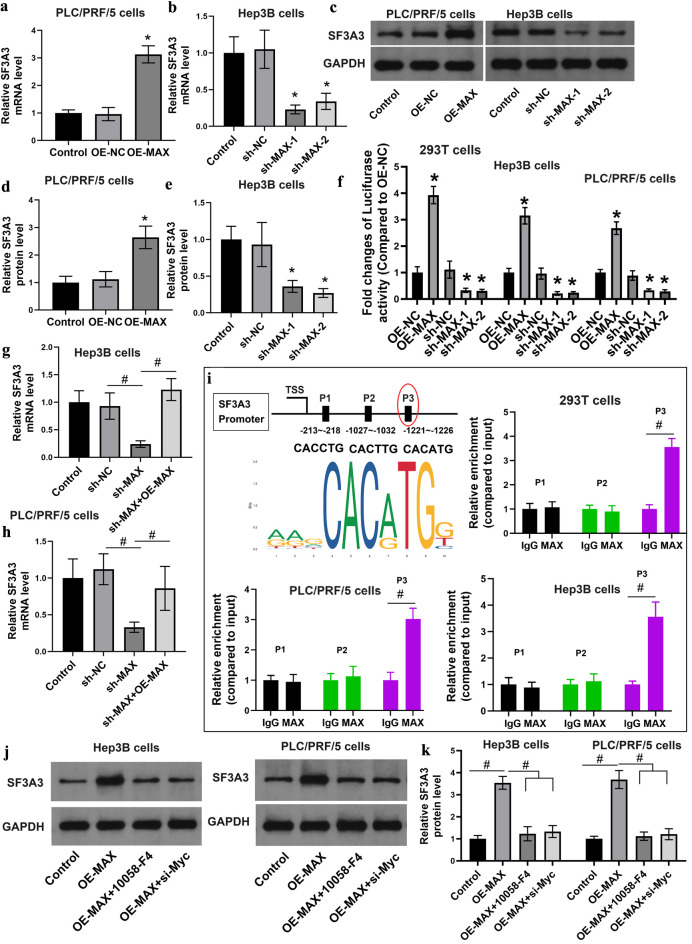
The transcriptional factor MAX enhanced the expression of SF3A3. (a)–(e) qRT-PCR and Western blot analyses of SF3A3 expression following MAX knockdown in Hep3B cells or overexpression in PLC/PRF/5 cells (*P < 0.05 compared to OE-NC or sh-NC). (f) Relative luciferase reporter activity of the SF3A3 promoter in 293T and Hep3B cells following MAX overexpression or knockdown (*P < 0.05 compared to OE-NC or sh-NC). (g) and (h) qRT-PCR evaluation of SF3A3 mRNA rescue by exogenous MAX-expression plasmid in sh-MAX-treated cells (^#^P < 0.05 between indicated groups). (i) ChIP-qPCR analysis of MAX binding enrichment at predicted E-box sites (P1–P3) within the SF3A3 promoter (^#^P < 0.05 between indicated groups). Western blot analysis (j) and quantification (k) of SF3A3 protein levels following co-treatment with OE-MAX and MYC inhibitor (10058-F4) or si-MYC. Data are mean ± SEM (n = 3) (^#^P < 0.05 between indicated groups). Data are presented as mean ± SEM, n = 3 independent experiments.

To assess the correlation between the expression levels of SF3A3 and MAX, PLC/PRF/5 cells were transfected with a plasmid construct to overexpress MAX, whereas Hep3B cells were transfected with an shRNA to knock down MAX. Following these treatments, we measured the mRNA and protein expression levels of SF3A3 in both cell lines. Compared with control cells, PLC/PRF/5 cells with overexpression of MAX had significantly higher mRNA and protein expression levels of SF3A3, whereas Hep3B cells with knockdown of MAX had notably lower mRNA and protein expression levels of SF3A3 (Fig. 6a–e).

To validate the interaction between the two genes and its functional implications, we conducted luciferase reporter assay on 293T and Hep3B cells. Overexpression of MAX significantly increased luciferase activity, suggesting that MAX enhanced the transcription of the SF3A3 promoter. On the contrary, when MAX was knocked down using shRNA, luciferase activity was markedly reduced, indicating that MAX is essential for the transcriptional activation of SF3A3 (Fig. 6f).

To investigate the regulatory effects of MAX on SF3A3 expression, we treated Hep3B and PLC/PRF/5 cells with shMAX, an shRNA designed to specifically knock down MAX. Subsequently, these cells were transfected with a MAX-expression plasmid to assess the potential rescue of SF3A3 mRNA expression. Treatment with shMAX resulted in a significant decrease in SF3A3 mRNA expression in both cell lines, validating that MAX is a positive regulator of SF3A3 transcription. When the MAX-expression plasmid was introduced into the shMAX-treated cells, we observed a notable increase in SF3A3 mRNA expression, which indicated that exogenous MAX could compensate for the reduced MAX expression caused by shMAX and restore the transcription of SF3A3 (Fig. 6g and h).

To investigate the molecular mechanism through which MAX regulates SF3A3 expression, we initially identified potential transcription factor-binding sites within the SF3A3 promoter using the JASPAR database. The human SF3A3 promoter region (chr1: 37,990,022–37,991,522, GRCh38) was retrieved from the Eukaryotic Promoter Database. Bioinformatic analysis revealed three putative binding sites for the transcription factor MAX, located at positions −213 to −218 (P1: 5'-CACCTG-3'), −1027 to −1032 (P2: 5'-CACTTG-3'), and −1221 to −1226 (P3: 5'-CACATG-3') relative to the transcription start site (Fig. 6i). To experimentally validate these bioinformatic predictions, we designed ChIP-qPCR primers flanking each of these three sites. As shown in Figure 6i, significant enrichment of MAX-bound DNA was observed specifically at site P3 in both Hep3B and PLC/PRF/5 cells (approximately 3.5-fold enrichment compared to IgG control, P < 0.001), while sites P1 and P2 showed no significant enrichment. This experimental result is consistent with the bioinformatic prediction that the P3 site, containing the perfect CACATG consensus, represents the most favorable binding configuration for MAX.

To explore whether MAX-mediated SF3A3 transcription is Myc-dependent, we performed Myc inhibition experiments (using Myc inhibitor 10058-F4 and Myc siRNA) to assess whether MAX-mediated SF3A3 transcription is Myc-dependent. As shown in Figure 6j and k, inhibition of Myc by 10058-F4 or si-Myc abrogated the MAX-induced upregulation of SF3A3 protein expression. This result suggests that MAX likely regulates SF3A3 transcription as part of a Myc-MAX heterodimeric complex, which is consistent with its known function and our ChIP data showing direct MAX binding to the SF3A3 promoter.

### Reversal of MAX-induced oncogenic phenotypes by SF3A3 knockdown *in vitro*

**Figure 7:**
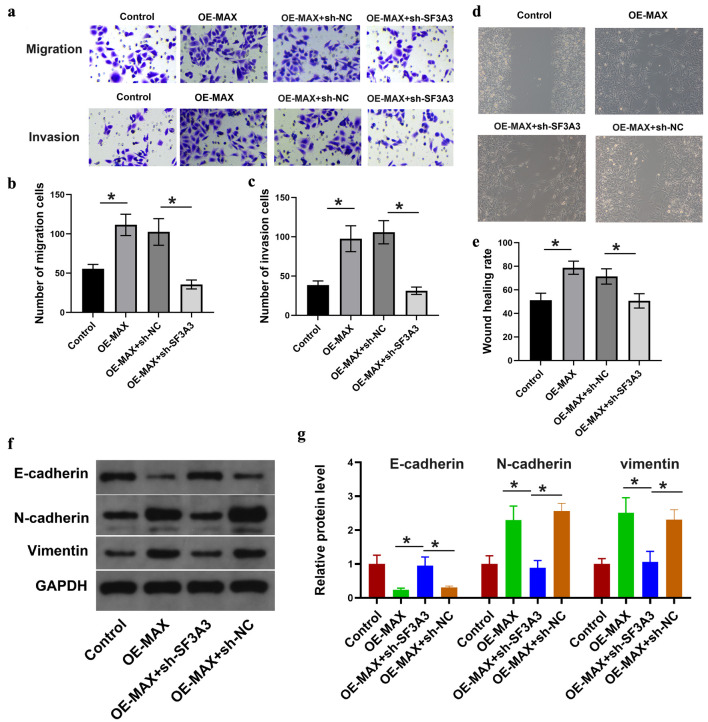
SF3A3 knockdown counteracted the effects of overexpressed MAX on liver cancer cell migration, invasion, and EMT. Functional assays evaluating cell migration (a, c), invasion (a, b), and wound healing (d, e) in PLC/PRF/5 cells co-transfected with MAX overexpression plasmid and sh-SF3A3. Data are presented as mean ± SEM, n = 3 independent experiments; *P < 0.05 between indicated groups. (f) and (g) Western blot analysis of EMT-related markers in the indicated co-transfected PLC/PRF/5 cells. Data are presented as mean ± SEM (n = 3). *P < 0.05 between indicated groups.

To assess the effects of SF3A3 knockdown on the oncogenic phenotypes induced by MAX overexpression in PLC/PRF/5 cells, we initially overexpressed MAX in PLC/PRF/5 cells and subsequently introduced an shRNA to specifically knock down SF3A3. These cells were evaluated for changes in the wound healing rate; migratory cell count; invasive cell count; and expression of EMT-related proteins, including E-cadherin, N-cadherin, and vimentin. The results showed that knockdown of SF3A3 counteracted the MAX-induced increase in the wound healing rate and the number of migratory and invasive cells, suggesting that the oncogenic effects of overexpressed MAX were at least partially dependent on SF3A3 (Fig. 7a–e). Moreover, Western blotting showed that knockdown of SF3A3 counteracted the MAX-induced changes in the expression of the three EMT-related proteins (Fig. 7f and g).

### SF3A3 knockdown reverses MAX-induced tumor growth in a xenograft mouse model of HCC

**Figure 8:**
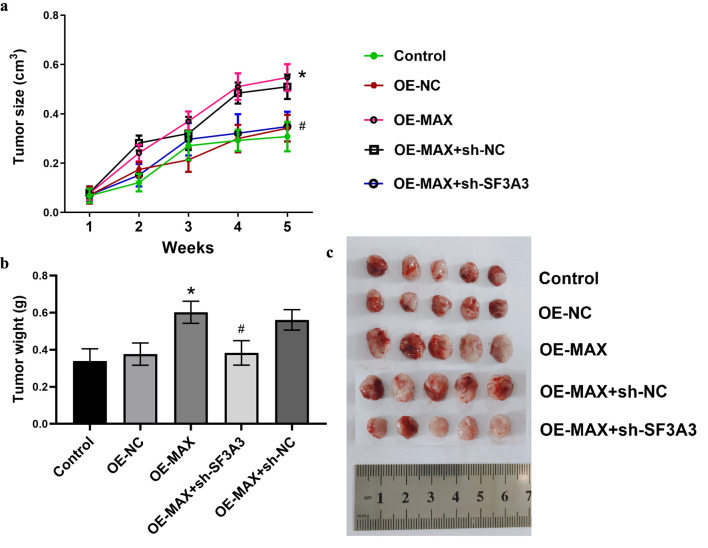
SF3A3 knockdown attenuates MAX-induced tumor growth *in vivo*. (a) Tumor growth curves of subcutaneous xenografts in nude mice injected with the indicated PLC/PRF/5 cell lines over 5 weeks. (b) Representative images of the excised tumors at the experimental endpoint. (c) Final tumor weights. Data are presented as mean ± SEM (n = 5). *P < 0.05 vs. Control group; ^#^P < 0.05 vs. OE-MAX group.

To investigate the impact of MAX and SF3A3 on tumorigenesis, we performed an *in vivo* experiment using a nude mouse model of HCC. PLC/PRF/5 cells with MAX overexpression, with either a non-targeting control or SF3A3 knockdown, were subcutaneously injected into nude mice. The MAX-overexpressing cells led to a notable increase in tumor growth, whereas knockdown of SF3A3 in these cells resulted in a significant reduction in tumor growth, effectively reversing the tumor-promoting effects of overexpressed MAX (Fig. 8).

## Discussion

HCC remains a leading cause of cancer-related mortality worldwide, and the identification of novel molecular drivers is critical for developing effective therapies [1, 2]. In this study, we uncovered a previously unrecognized regulatory axis involving the transcription factor MAX and the splicing factor SF3A3 that promotes HCC progression. Our bioinformatic analysis and clinical sample validation results are in line with recent reports implicating splicing factor dysregulation in cancer [10, 11] and expand the understanding of MAX as a key oncogenic partner beyond its classic role in the Myc network [17–19]. While our clinical validation in patient samples yielded significant correlations, the sample size remains relatively modest. Therefore, these clinical findings should be considered preliminary, and future validation in larger, multi-center HCC cohorts is necessary to confirm the prognostic value of the MAX/SF3A3 axis. Previous studies have established the oncogenic role of SF3A3 in various cancers; however, its upstream transcriptional regulation has remained poorly understood. By identifying MAX as a direct transcriptional activator of SF3A3, our study extends prior work by linking core spliceosome machinery to the well-known MYC/MAX oncogenic transcriptional network, providing a new upstream target for therapeutic intervention.

Mechanistically, we showed for the first time that MAX directly binds to the SF3A3 promoter and enhances its transcription. This transcriptional regulation is consistent with the known function of MAX as a DNA-binding protein that forms heterodimers with Myc or MXD family members to modulate gene expression [18, 19]. The identification of SF3A3 as a direct transcriptional target of MAX adds a new dimension to the Myc-MAX regulatory network, linking it to the splicing machinery. Recent studies have shown that SF3A3 is upregulated by overactivated Myc signaling and contributes to metabolic reprogramming and stemness in breast cancer [16]. To explore whether MAX-mediated SF3A3 transcription is Myc-dependent, we performed Myc inhibition experiments (using Myc inhibitor 10058-F4 and Myc siRNA). This finding is critical for defining the molecular nature of the interaction. While there is an intriguing possibility of a physical interaction involving MAX, MYC, and SF3A3 proteins together, our combined data strongly support a transcriptional regulation model. Since MAX typically heterodimerizes with MYC to bind E-box motifs (CACATG) and activate transcription, we conclude that the MYC-MAX heterodimer acts cooperatively at the SF3A3 promoter to drive its expression, rather than forming a ternary protein complex with SF3A3. This establishes SF3A3 as a direct downstream target of the oncogenic MYC-MAX network in HCC. To our knowledge, this study provides the first demonstration of direct MAX binding to the SF3A3 promoter in HCC, expanding the current understanding of the MYC/MAX network beyond its classical targets. The MYC-MAX heterodimer is canonically recognized as a master transcriptional activator driving cell cycle and metabolism. However, the dynamics of this network are highly context-dependent, as MAX can also homodimerize or interact with MAD/MNT family proteins to repress transcription. In our study, while ChIP-qPCR confirmed direct MAX occupancy at the P3 E-box of the SF3A3 promoter, and MYC inhibition successfully attenuated SF3A3 expression, we did not directly assess MYC binding. Therefore, while our data strongly support a functional dependence on MYC, the hypothesis of cooperative MYC-MAX heterodimer-driven regulation of SF3A3 should be interpreted cautiously until direct MYC co-occupancy is experimentally validated in future ChIP assays.

As a core component of the U2 snRNP complex, SF3A3 governs precursor mRNA splicing—a fundamental biological process frequently hijacked in cancer. Recent literature highlights that splicing factors exert their oncogenic effects not merely by altering global gene expression, but by driving specific transcript isoform switches that favor tumor progression. For instance, the alternative splicing of the CD44 gene yields the standard isoform (CD44s) and multiple variant isoforms (CD44v), each contributing differently to cancer progression. The variant isoforms, in particular, have been implicated in promoting EMT, a process that enhances cancer cell invasiveness and metastatic potential [23]. Specific variants, such as CD44v6, are regulated by splicing factors like SFPQ, which promote lung cancer malignancy by enhancing stemness, proliferation, and metastasis [24]. Splicing shifts in PKM and BCL2L1 are critical for metabolic reprogramming and evasion of apoptosis, respectively. One relevant study investigates the role of polypyrimidine tract binding (PTB) protein in the alternative splicing of PKM, specifically the switch from PKM1 to PKM2, which is crucial for aerobic glycolysis in keloid fibroblasts [25]. This switch is a hallmark of the Warburg effect, a metabolic phenotype commonly observed in cancer cells. Differential splicing responsiveness among anti-apoptotic BCL2 family genes dictates the selective cytotoxicity of SF3b-targeting modulator E7107. Since BCL2L1 resists E7107-induced splicing alteration, this agent specifically elicits apoptosis in BCL2A1-dependent melanoma cells and MCL1-dependent NSCLC cells [26]. Pladienolide B-mediated SF3B1 inhibition markedly modulates diverse glioblastoma malignant behaviors, such as inhibited proliferation, migration, tumor sphere formation, vascular endothelial growth factor (VEGF) release and tumor initiation, alongside elevated apoptosis. Such effects are likely attributed to inactivation of the AKT/mTOR/β-catenin axis and aberrant BCL2L1 splicing homeostasis [27]. Although our study firmly establishes the upstream regulation of SF3A3 by MAX, the precise downstream splicing events orchestrated by SF3A3 in HCC remain to be elucidated. Future investigations mapping the SF3A3-dependent splicing landscape in HCC will be crucial to identifying the exact oncogenic isoforms responsible for the observed malignant phenotypes.

A recent study confirmed that SF3A3 drives tumor progression in HCC via the PI3K/AKT pathway [28]. SF3A3 was identified as a valuable diagnostic indicator and independent prognostic predictor for HCC. At the molecular level, knockdown of SF3A3 in HCC cells significantly attenuated cellular proliferation, migratory capacity, PI3K/AKT signaling activity, and the expression of EMT markers, which further confirmed its pivotal role in driving the malignant progression of HCC [28]. Our results extend this concept to HCC and establish a direct regulatory relationship.

Functional assays demonstrated that MAX promotes HCC cell proliferation, migration, invasion, and EMT, and these effects are largely mediated by SF3A3. These observations are consistent with the emerging role of alternative splicing in cancer progression [12, 29]. For instance, aberrant splicing events regulated by SF3A3 may generate isoforms of genes involved in cell adhesion, cytoskeleton dynamics, and signaling pathways that favor metastasis [15, 30]. Given SF3A3’s role as a core component of the U2 snRNP complex [15], it is plausible that its dysregulation leads to the production of oncogenic protein variants that promote tumor growth and invasion. However, the specific splicing events and downstream isoforms responsible for these phenotypes remain to be identified. Future studies employing RNA sequencing coupled with bioinformatic splicing analysis (e.g., rMATS, MISO) are warranted to comprehensively profile SF3A3-dependent splicing changes in HCC cells. Candidate splice variants with potential oncogenic functions (such as isoforms of genes involved in EMT, migration, or proliferation) should be validated by RT-PCR and functionally tested through isoform-specific overexpression or knockdown experiments. Additionally, CLIP-seq (crosslinking and immunoprecipitation followed by sequencing) could identify direct RNA targets of SF3A3, providing mechanistic insight into its splicing regulatory network. Such investigations would clarify whether the oncogenic effects of SF3A3 are mediated by a few critical splice variants or by widespread splicing dysregulation.

The upregulation of mesenchymal markers (N-cadherin, vimentin) and downregulation of E-cadherin upon MAX overexpression further support the notion that the MAX-SF3A3 axis drives EMT, a critical step in HCC dissemination [31]. It is well established that EMT promotes tumor cell migration and invasion by reducing epithelial cell-cell adhesion and increasing cellular motility. While our data indicate that the MAX/SF3A3 axis alters the expression of classical epithelial and mesenchymal markers (E-cadherin, N-cadherin, and vimentin) consistent with an EMT-like phenotype, it should be noted that key EMT-inducing transcription factors (such as SNAIL, ZEB, or TWIST) were not assessed in this study. Thus, further investigation is required to fully characterize the scope of EMT regulation by this axis. Additionally, EMT and alternative splicing are deeply interconnected with the acquisition of cancer stem cell (CSC) properties, which contribute to therapeutic resistance and tumor recurrence in HCC. Tumor plasticity, characterized by EMT and the acquisition of CSC properties, is deeply intertwined with aberrant alternative splicing. Functionally, EMT promotes metastasis by reducing epithelial cell-cell adhesion and enhancing cytoskeletal motility. Our findings indicate that the MAX/SF3A3 axis promotes migration, invasion, and alters classical EMT markers (E-cadherin, N-cadherin, and vimentin). However, we recognize that true tumor plasticity extends beyond these basic markers. The acquisition of an EMT-like state is frequently accompanied by enhanced stemness, which drives therapeutic resistance and HCC recurrence. Because our study did not evaluate key EMT-inducing transcription factors (e.g., SNAIL, ZEB, TWIST) or specific stemness parameters (e.g., CD133 expression, sphere-forming capacity), the full extent to which the MAX/SF3A3 axis drives tumor plasticity remains partially characterized. Although our findings demonstrate that the MAX/SF3A3 axis promotes migration, invasion, and an EMT-like phenotype, it remains unclear whether this pathway also enhances CSC properties. The absence of stemness-associated analyses (e.g., assessing PROM1/CD133, EpCAM expression, or sphere formation capacity) is an important limitation of this study. Future studies incorporating these assays will be crucial to determining whether SF3A3 drives tumor plasticity and stemness in HCC.

An important consideration arising from our study is the novelty of the MAX-SF3A3 regulatory axis. To our knowledge, this is the first report demonstrating that MAX directly binds to the promoter of SF3A3 to regulate its transcription in HCC. While SF3A3 has been previously identified as a downstream target of overactivated Myc signaling in breast cancer [16], the mechanistic details of its transcriptional regulation remained unexplored. Our study fills this gap by demonstrating that MAX, the canonical heterodimerization partner of Myc, directly binds to the SF3A3 promoter and activates its transcription. The preferential binding of MAX to the P3 site in the SF3A3 promoter, over P1 and P2, is explained by MAX’s well-characterized DNA-binding specificity. As a basic helix-loop-helix leucine zipper protein, MAX binds the consensus E-box sequence CACATG. The P3 site perfectly matches this consensus, enabling optimal hydrogen bonding and hydrophobic interactions. The concordance between our bioinformatic predictions, ChIP-qPCR results, and published structural data strongly suggests P3 as a functional MAX response element, providing a robust mechanistic foundation for MAX-mediated SF3A3 regulation in HCC. This finding expands the current understanding of the Myc-MAX network beyond its classical role in regulating proliferation and metabolism [17–19], revealing a previously unrecognized link between this oncogenic transcription factor complex and the splicing machinery. The absence of prior reports on this interaction is not surprising given that most studies have focused on Myc as the primary transcriptional activator, with MAX often viewed merely as a passive dimerization partner [32, 33]. Our results challenge this paradigm by demonstrating that MAX itself plays an active and direct role in gene regulation, at least for the SF3A3 locus. Furthermore, the functional validation through rescue experiments establishes that the oncogenic effects of MAX in HCC are mediated, at least in part, through SF3A3. Collectively, these multiple lines of evidence (ChIP-qPCR, luciferase reporter assays with site-directed mutagenesis, and functional rescue experiments) provide support for our hypothesis of the MAX-SF3A3 regulatory axis in HCC.

Our findings also align with studies showing that splicing factors can be targeted therapeutically [13, 34]. The SF3A3-containing spliceosome complex has been explored as a drug target in cancers, with compounds like pladienolide B showing efficacy [16]. However, whether such inhibitors can specifically disrupt the MAX-SF3A3 interaction or downstream splicing events in HCC remains to be investigated. Given the poor prognosis associated with high MAX and SF3A3 expression, targeting this axis may offer a novel therapeutic strategy, particularly for patients with advanced HCC who have limited options [5, 6].

Despite the strengths of our study, several limitations should be acknowledged. First, the precise molecular mechanism by which SF3A3 modulates specific splicing events to promote HCC malignancy warrants deeper investigation. RNA-seq and splicing-sensitive microarray analyses could identify global splicing changes induced by MAX-SF3A3 dysregulation. Second, while we demonstrated a direct transcriptional regulation, the potential involvement of other MAX-interacting partners (e.g., MNT, MGA) in modulating SF3A3 expression cannot be excluded and merits further study [19]. Third, our *in vivo* model used subcutaneous xenografts, which do not fully recapitulate the tumor microenvironment and metastatic process; orthotopic or genetically engineered mouse models would provide more clinically relevant insights. Finally, the clinical utility of MAX and SF3A3 as biomarkers or therapeutic targets requires validation in larger, multi-center cohorts and exploration of combination therapies, such as with immune checkpoint inhibitors [34, 35]. Furthermore, as a core component of the spliceosome, SF3A3 is known to exert oncogenic effects by regulating alternative splicing (AS) events. Dysregulated splicing contributes to cancer progression through specific isoform switches, such as CD44 (promoting EMT and stemness), PKM (driving metabolic reprogramming), and BCL2L1 (regulating apoptosis). Although our study firmly establishes the MAX/SF3A3 regulatory axis in HCC, the specific downstream splicing targets of SF3A3 were not investigated. This represents a limitation of the current study. Future research should focus on identifying the specific transcript isoforms modulated by the MAX/SF3A3 axis using RNA-seq and alternative splicing analysis to fully elucidate the downstream mechanisms.

In conclusion, the MAX-SF3A3 axis serves as an important regulatory mechanism in HCC. The overexpression of MAX and SF3A3 in HCC tissues and their association with poor patient survival highlight the potential of the two genes as therapeutic targets for HCC. Targeting the MAX-SF3A3 axis represents a novel strategy for the treatment of HCC. As research on the pathological mechanisms of HCC continues, the findings of this study offer promising avenues for further research into the MAX-SF3A3 axis and its use as a therapeutic target in clinical settings. This study identifies a novel MAX-SF3A3 regulatory axis that drives HCC progression by enhancing cell proliferation, migration, invasion, and EMT. Our results provide a mechanistic link between transcriptional control and splicing dysregulation in HCC, and highlight the potential of targeting this axis as a therapeutic approach. Future studies aimed at dissecting the downstream splicing network and developing specific inhibitors will be crucial for translating these findings into clinical applications.

## Supplementary Material

Suppl 1 ChIP-qPCR primer sequences and amplicon details for the SF3A3 promoter.

## Data Availability

All data necessary to reproduce the analyses presented in this study are available from the corresponding authors upon reasonable request.

## Abbreviations

AKT: protein kinase B
ANOVA: analysis of variance
BEGM: bronchial epithelial growth medium
CCK-8: cell counting kit-8
cDNA: complementary DNA
ChIP: chromatin immunoprecipitation
CLIP-seq: crosslinking and immunoprecipitation followed by sequencing
DMEM: Dulbecco’s modified Eagle medium
EMT: epithelial–mesenchymal transition
FBS: fetal bovine serum
GAPDH: glyceraldehyde-3-phosphate dehydrogenase
GEPIA: Gene Expression Profiling Interactive Analysis
HBV: hepatitis B virus
HCC: hepatocellular carcinoma
IgG: immunoglobulin G
HCC: liver hepatocellular carcinoma (TCGA project code)
MAX: MYC associated factor X
MYC: MYC proto-oncogene (c-Myc)
OE-NC: overexpression negative control
PBS: phosphate-buffered saline
PI3K: phosphatidylinositol 3-kinase
PWM: position weight matrix
qPCR: quantitative polymerase chain reaction
qRT-PCR: quantitative reverse transcription polymerase chain reaction
RNA-seq: RNA sequencing
SDS-PAGE: sodium dodecyl sulfate–polyacrylamide gel electrophoresis
SEM: standard error of the mean
SF3A3: splicing factor 3a subunit 3
sh-NC: short hairpin RNA negative control
shRNA: short hairpin RNA
siRNA: small interfering RNA
SPF: specific pathogen-free
TCGA-HCC: The Cancer Genome Atlas Liver Hepatocellular Carcinoma
