# Supplementary material for: Transcription Factor MAX Regulates Liver Cancer Cell Growth, Migration, Invasion, and Epithelial–Mesenchymal Transition by Promoting SF3A3 Expression
Source: World J Oncol. 2026 Jun 25;17(4):556–71. doi: 10.14740/wjon2783 (PMC13375434; doi:10.14740/wjon2783)
Supplement: Suppl 1 — ChIP-qPCR primer sequences and amplicon details for the SF3A3 promoter. [file wjon-17-04-556-s001.docx]

**Suppl 1.** ChIP-qPCR Primer Sequences and Amplicon Details for the SF3A3 Promoter

| **Target Site** | **Primer Sequence (5’–3’)** | **Amplicon Size (bp)** | **Position Relative to TSS** | **Predicted sequences** |
| --- | --- | --- | --- | --- |
| **Site P1** | **F:** GTGCCCCGGGACTTAAGAAA  **R:** GAGGCATCTGCGAACCTCTC | 104 bp | -213 to -218 | CACCTG |
| **Site P2** | **F:** TGAGGACGCAGCTTTCAGC  **R:** CTGAGAAAGACGCTGAGGTCC | 70 bp | -1027 to -1032 | CACTTG |
| **Site P3** | **F:** ACTTGAGGACGCAGCTTTCA **R:** CCACCAGCAAAACCCCACTA | 104 bp | -1221 to -1226 | CACATG |
